# Rapid complete blood count and C-reactive protein determination with the Horiba Microsemi analyzer: the experience in neonatal intensive care unit of Careggi University Hospital

**DOI:** 10.1007/s00431-024-05695-0

**Published:** 2024-08-15

**Authors:** Francesca Nencini, Alessandro Bonari, Francesca Romano, Sara Ciullini Mannurita, Alessandra Mongia, Maria Garieri, Carlo Dani, Orazio Manzo, Maria Pontieri, Alessandra Fanelli

**Affiliations:** 1 General Laboratory, Careggi University Hospital, Florence, Italy; 2 Division of Neonatology and Neonatal Intensive Care Unit, Careggi University Hospital, Florence, Italy; 3 Department of Neurosciences, Psychology, Drug, Research and Child Health (NEUROFARBA), University of Florence, Viale Pieraccini, 6, Florence, Italy

**Keywords:** Complete blood count, Hematology analyzer, C-reactive protein, Performance comparison, Microsystem, Newborn

## Abstract

Microsystems represent an alternative but proficient approach of analysis outside the laboratory, and their use could help in reducing the impact of pre-analytical errors, in particular in challenging newborn samples. The study purpose is to compare the Horiba Microsemi CRP LC-767G system for rapid 3-part complete blood count (CBC) and C-reactive protein (CRP) determination with the laboratory reference systems (respectively Sysmex XN-9100™ and Roche Cobas® c702) in samples of adult patients and newborns hospitalized in the neonatal intensive care unit (NICU) samples. The comparison between the analyzers was performed through Passing-Bablok regression analysis and Bland–Altman plot. One hundred eighty-three blood samples were analyzed. The regression analysis results, performed in the newborn (*n* = 70) and in adult (*n* = 113) populations, showed a good agreement between the instruments. The evaluation of the Bland–Altman plots showed comparable values of bias < 10% for most of the parameters, but not for MPV, lymphocyte, and monocyte count. *Conclusion*: The comparison between the Microsemi CRP LC-767G system and the laboratory instrumentations demonstrated comparable results. The Microsemi CRP LC-767G system provides reliable analytical data and faster turnaround time, particularly useful in NICU.

**Table:** 

**What is Known:** *• Microsystems for point-of-care testing (POCT) represent an alternative but proficient approach of analysis outside the laboratory, in order to perform a rapid, safe, and exhaustive evaluation for critical patients’ management, acting as a valid support for treatment in acute care.*
**What is New:** *• The Microsemi CRP LC-767G system can represent an alternative but effective testing approach outside the laboratory, particularly in NICU, to reduce the impact of pre-analytical errors on newborn samples.*

**Supplementary Information:**

The online version contains supplementary material available at 10.1007/s00431-024-05695-0.

## Introduction

Microsystems for point-of-care testing (POCT) represent an alternative but proficient approach of analysis outside the laboratory, in order to perform a rapid, safe and exhaustive evaluation for critical patients’ management, acting as a valid support for treatment in acute care [1]. Complete blood count (CBC) and C-reactive protein (CRP) determinations in blood samples are considered the most effective biomarkers for inflammatory diseases such as bacterial infections [2, 3]. Unfortunately, blood samples may not be suitable due to pre-analytical and technical variables (accounting for approximately 60–70% of all blood sampling errors), associated with procedural skills of nurses and laboratory assistants [4–7], especially in critical patients, such as newborns and those admitted to intensive care units.

The use of a microsystem could improve the performance of analysis on biological samples, by reducing turnaround time, costs, the involvement of laboratory personnel and especially the required sample volume, but quality standards for this application must be verified by laboratory specialists.

In accordance with method comparison protocols defined by international guidelines (document EP09-A2 of the Clinical and Laboratory Standards Institute, CLSI) and adopted in this study, the results of blood count parameters provided by the Horiba Microsemi CRP LC-767G instrumentation and the Sysmex XN-9100™ were compared, using venous and arterial blood samples taken from adult and newborn patients. The LC-767G instrumentation also provides simultaneous CRP measurements, which were compared to those of the Roche Cobas® c702 system on a series of adult samples.

The purpose of this study is to evaluate the performance of the Microsemi CRP LC-767G analyzer for its use in the neonatal intensive care unit (NICU).

## Material and methods

### Study design

The Microsemi CRP LC-767G (Horiba Ltd, Kyoto, Japan) requires only 60 µl (18 µl for determinations and 42 µl for dead volume) of whole blood for simultaneous measurement of CBC and CRP, and rapidly provides these data in approximately 4 min. It is equipped with the electrical resistance method for CBC with 3-Diff leukocyte classification: neutrophilic, eosinophilic and basophilic granulocytes (GRA); lymphocytes (LYM); monocytes (MON). CRP is measured by the latex immune turbidimetry method, after prompt hemolyzation of EDTA-2 K anticoagulated whole blood. The obtained value is converted into plasma concentration according to the Hematocrit (HCT %) of the respective sample, finally providing the result as ‘whole blood CRP’.

A method comparison study was undertaken to compare the Microsemi CRP LC-767G system with Central Laboratory reference analyzers: the Sysmex XN-9100^TM^ system (Sysmex Corporation, Kobe, Japan) for CBC and the Cobas® c702 (Roche Diagnostics, USA) for CRP. The study design was based on the methods outlined in CLSI H20-A2, CLSI H26-A2 and CLSI EP09-A3.

Whole blood samples were collected from newborn patients (0 days to 2 months old) admitted to the NICU of the Careggi University Hospital (Florence) and from adults (> 22 years old), to have a group of samples from the Horiba Microsemi CRP LC-767G system validated population. Newborn samples were capillary whole blood samples, collected in 0.5-ml microtubes with EDTA-2 K anticoagulant (supplied by the instrument manufacturer Horiba), while adult samples were venous whole blood samples, collected in standard EDTA-2 K collection tubes (Becton Dickinson, Franklin Lakes, NJ, USA).

CRP determination was performed only on samples collected from adult subjects and a comparison between serum collection tube and EDTA-2 K collection tube was performed: samples collected in EDTA-2 K tubes and processed with the Microsemi CRP LC-767G system were then centrifuged and plasma analyzed with the Roche Cobas® c702, as well as the corresponding serum samples.

The analyses were carried out on the residual material within 2 h of sampling and no later than 1 h between the two instruments being compared.

The study included both normal and pathological samples to assess the Microsemi CRP LC-767G system performance across the entire analytical measuring range and around medical decision points.

For CBC, only certified parameters were considered: WBC, RBC, HGB, HCT, MCV, RDW, MCH, MCHC, PLT, MPV. The comparison relating to the parameters GRA, LYM, MON, being the differential count of the leukocyte populations not yet validated for the Microsemi CRP LC-767G instrument, was carried out for experimental purposes only. To compare the WBC differential count, we regarded as GRA the sum of neutrophils, eosinophils and basophils, measured by the reference instrumentation.

The samples’ loading and analysis on the instruments were carried out according to the manufacturer’s specifications.

### Statistical analysis

A Passing-Bablok regression analysis was performed for each parameter, after excluding any invalidated result by the Microsemi CRP LC-767G system or the reference analyzer. For each regression analysis, the slope, the intercept and the 95% two-sided confidence interval (CI) around the slope, as well as the correlation coefficient, were calculated using Bootstrap Method. The overall bias in terms of percentage was calculated as the values on the axis [(method A – method B) / mean)] vs. the mean of the two measurements (Bland–Altman plots). In the supplementary information, plots showing the mean difference also in terms of metric unit is added in order to estimate the numeric distance of the microsystem measurements with the reference methods (Sysmex XN and COBAS 702 for CBC and CRP respectively). Statistical analysis was performed using the online software https://bahar.shinyapps.io/method_compare/ [8, 9].

## Results

### Study population

The comparison in terms of accuracy between the Microsemi CRP LC-767G and the Sysmex XN-9100™ system was performed testing a total of 183 residual whole blood clinical samples: 113 (61.7%) from adult patients and 70 (38.3%) from newborn patients (Tables 1 and 2). The entire analyzed population was equally distributed between males and females (50.3% vs 49.7% respectively).

**Table 1:** Characteristics of adult study population

Sample number	Age (years)	Sex	Selection criteria
14	80	F	Healthy donor
19	57	M	Healthy donor
27	37	F	Healthy donor
28	50	F	Healthy donor
29	45	F	Healthy donor
30	33	M	Healthy donor
31	59	M	Healthy donor
32	55	F	Healthy donor
33	56	F	Healthy donor
34	80	F	Healthy donor
35	47	F	Healthy donor
36	77	M	Healthy donor
37	68	M	Healthy donor
38	70	F	Healthy donor
39	41	M	Healthy donor
40	47	M	Healthy donor
41	65	F	Healthy donor
42	66	F	Healthy donor
43	29	M	Healthy donor
44	66	F	Healthy donor
8	74	M	Leukocytopenia
10	77	M	Leukocytopenia
96	66	M	Leukocytopenia
103	59	M	Leukocytopenia
50	74	M	Leukocytopenia
73	34	M	Leukocytopenia-thrombocytopenia
76	57	M	Leukocytopenia-thrombocytopenia
64	49	F	Leukocytopenia-thrombocytopenia-low hemoglobin
79	68	F	Leukocytopenia-thrombocytopenia-low hemoglobin
80	66	M	Leukocytopenia-thrombocytopenia-low hemoglobin
81	51	F	Leukocytopenia-thrombocytopenia-low hemoglobin
88	64	M	Leukocytopenia-thrombocytopenia-low hemoglobin
94	72	M	Leukocytopenia-thrombocytopenia-low hemoglobin
100	62	F	Leukocytopenia-thrombocytopenia-low hemoglobin
7	87	F	Leukocytosis
18	80	F	Leukocytosis
25	71	M	Leukocytosis
26	75	F	Leukocytosis
87	54	F	Leukocytosis
67	68	M	Leukocytosis
70	72	F	Leukocytosis
71	59	F	Leukocytosis
75	26	F	Leukocytosis
82	88	F	Leukocytosis
83	64	M	Leukocytosis
84	75	M	Leukocytosis
92	72	F	Leukocytosis
95	79	M	Leukocytosis
97	53	M	Leukocytosis
102	82	F	Leukocytosis
6	67	F	Leukocytosis-low hemoglobin
16	67	F	Leukocytosis-low hemoglobin
51	76	M	Leukocytosis-low hemoglobin
85	75	M	Leukocytosis-low hemoglobin
86	70	F	Leukocytosis-low hemoglobin
89	75	F	Leukocytosis-low hemoglobin
68	66	M	Leukocytosis-thrombocytopenia
1	83	F	Low hemoglobin
4	76	M	Low hemoglobin
11	80	F	Low hemoglobin
12	70	F	Low hemoglobin
13	78	M	Low hemoglobin
17	82	F	Low hemoglobin
20	78	M	Low hemoglobin
21	80	M	Low hemoglobin
22	67	F	Low hemoglobin
23	77	F	Low hemoglobin
24	54	M	Low hemoglobin
72	69	M	Low hemoglobin
77	56	F	Low hemoglobin
2	78	M	Low hemoglobin
15	70	M	Low hemoglobin
5	77	M	Low hemoglobin
91	53	F	Low hemoglobin-leukocytopenia-thrombocytopenia
3	85	M	Low hemoglobin-thrombocytosis
9	73	M	Thrombocytopenia
69	81	M	Thrombocytopenia
46	63	M	Thrombocytopenia-leukocytopenia
47	82	F	Thrombocytopenia-low hemoglobin
55	102	F	Thrombocytopenia-low hemoglobin
58	72	F	Thrombocytopenia-low hemoglobin
59	64	F	Thrombocytopenia-low hemoglobin
65	75	F	Thrombocytopenia-low hemoglobin
48	52	F	Thrombocytopenia-low hemoglobin-leukocytopenia
53	66	M	Thrombocytopenia-low hemoglobin-leukocytopenia
54	65	M	Thrombocytopenia-low hemoglobin-leukocytopenia
56	45	F	Thrombocytopenia-low hemoglobin-leukocytopenia
57	61	M	Thrombocytopenia-low hemoglobin-leukocytopenia
60	66	F	Thrombocytopenia-low hemoglobin-leukocytopenia
62	65	F	Thrombocytopenia-low hemoglobin-leukocytopenia
61	53	M	Thrombocytopenia-low hemoglobin-leukocytosis
45	74	F	Thrombocytosis
52	61	F	Thrombocytosis
63	50	M	Thrombocytosis
66	70	M	Thrombocytosis
104	53	F	Thrombocytosis
105	32	F	Thrombocytosis
109	68	F	Thrombocytosis
78	74	F	Thrombocytosis
90	27	F	Thrombocytosis
74	63	M	Thrombocytosis-leukocytosis
49	79	F	Thrombocytosis-leukocytosis-low hemoglobin
106	51	F	Thrombocytosis-leukocytosis-low hemoglobin
111	33	M	Thrombocytosis-leukocytosis-low hemoglobin
113	31	M	Thrombocytosis-leukocytosis-low hemoglobin
93	20	F	Thrombocytosis-leukocytosis-low hemoglobin
98	38	M	Thrombocytosis-low hemoglobin
99	73	F	Thrombocytosis-low hemoglobin
101	83	M	Thrombocytosis-low hemoglobin
107	80	M	Thrombocytosis-low hemoglobin
108	48	F	Thrombocytosis-low hemoglobin
110	65	M	Thrombocytosis-low hemoglobin
112	74	F	Thrombocytosis-low hemoglobin

**Table 2:** Characteristics of newborn study population

Sample number	Age (days)	Sex	Microsemi CRP results	Pre-analytical evaluation	XN-9100™ results
1	1	F	Done		Done
2	11	M	Done		Done
3	1	F	Done		Done
4	1	F	Done		Done
5	1	M	Done		Done
6	1	M	Done		Done
7	1	F	Done		Done
8	1	M	Done		Done
9	1	M	Done		Done
10	1	M	Done		Done
11	1	M	Done	Presence of clots	Not done
12	1	M	Done		Done
13	7	M	Done		Done
14	1	F	Done		Done
15	1	M	Done	Presence of clots	Not done
16	1	M	Done	Presence of clots	Not done
17	1	F	Done		Done
18	1	M	Done	Presence of clots	Not done
19	3	M	Done		Done
20	3	F	Done		Done
21	3	F	Done		Done
22	1	M	Done		Done
23	1	F	Done		Done
24	1	M	Done		Done
25	2	F	Done		Done
26	3	F	Done		Done
27	1	F	Done		Done
28	1	F	Done		Done
29	1	M	Done	Presence of clots	Not done
30	1	M	Done		Done
31	2	M	Done		Done
32	5	F	Done	Presence of clots	Not done
33	3	M	Done	Presence of clots	Not done
34	3	F	Done		Done
35	10	M	Done	Presence of clots	Not done
36	1	F	Done		Done
37	1	M	Done		Done
38	6	F	Done		Done
39	1	M	Done		Done
40	5	M	Done		Done
41	7	F	Done		Done
42	6	M	Done	Insufficient sample volume	Not done
43	1	M	Done	Presence of clots	Not done
44	2	M	Done	Presence of clots	Not done
45	2	M	Done	Presence of clots	Not done
46	7	M	Done		Done
47	9	F	Done		Done
48	60	F	Done		Done
49	2	M	Done		Done
50	2	F	Done	Presence of clots	Not done
51	15	M	Done		Done
52	11	F	Done		Done
53	1	M	Done	Presence of clots	Not done
54	1	F	Done	Insufficient sample volume	Not done
55	2	M	Done		Done
56	2	M	Done		Done
57	2	M	Done		Done
58	3	M	Done	Presence of clots	Not done
59	1	F	Done		Done
60	1	M	Done	Presence of clots	Not done
61	1	F	Done		Done
62	4	F	Done	Insufficient sample volume	Not done
63	2	F	Done		Done
64	1	F	Done	Presence of clots	Not done
65	5	F	Done		Done
66	8	M	Done		Done
67	1	F	Done		Done
68	6	F	Done		Done
69	1	M	Done		Done
70	1	M	Done		Done

Sixteen newborn samples (22.9%), initially uncoagulated and successfully analyzed with the Microsemi CRP LC-767G system, were subsequently coagulated at the time of analysis using the reference analyzer. These samples were excluded from the study (Tables 1 and 2).

Three newborn samples (4.3%), tested with the Microsemi CRP LC-767G system, were not analyzed with the Sysmex XN-9100^TM^ due to insufficient sample volume. These samples were excluded from the study (Tables 1 and 2).

To summarize, we analyzed 51 newborn patients (of which 39 with also leukocyte differential count) and 113 adult patients.

The CRP determinations on the Microsemi CRP LC-767G were performed on all adult samples (*n* = 113), while the comparison between serum collection tube and EDTA-2 K collection tube determinations was performed on the Roche Cobas® c702 on 25 samples.

### Method comparison: CBC results

**Fig. 1:**
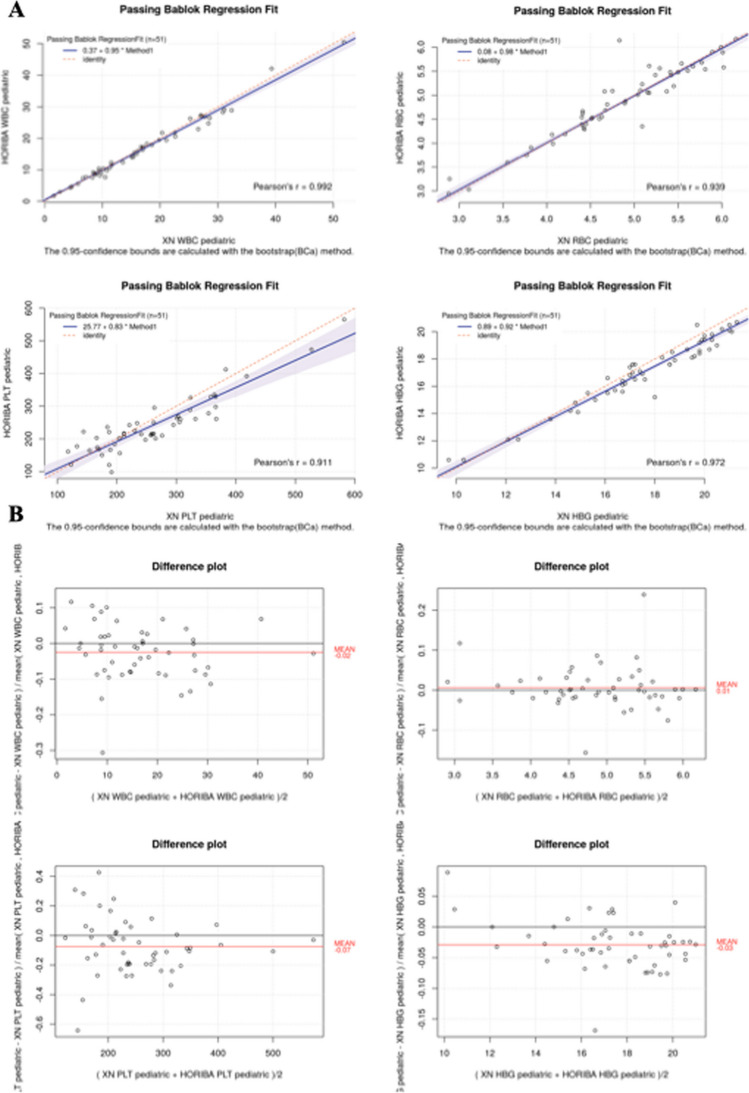
CBC regression analysis and bias plot in newborn population. Results of the method comparison study between the Microsemi CRP LC-767G and the Sysmex XN^TM^ hematology analyzers. Graphs indicate Pearson correlation, slope and intercept (**A**), and bias plots (**B**) for the main parameters. WBC, white blood cells; RBC, red blood cells; PLT, platelets; HGB, hemoglobin

Considering the newborn population, the regression analysis showed a good agreement between the Microsemi CRP LC-767G and the Sysmex XN-9100^TM^ system (Fig. 1A and Table 3). It was observed: high correlation (*r* ≥ 0.90) for the parameters WBC, RBC, PLT, HGB, HCT, granulocytes, lymphocytes, monocytes; moderate correlation (0.75 < *r* < 0.90) for MCV, RDW and MCH; no correlation for MCHC and MPV.

**Table 3:** Summary of the method comparison study in newborn population

Measurand	N	Results range	Correlation coefficient	Slope	Intercept	Mean bias Desirable limits for inaccuracy (%)
			**(*r*)**	**(95% CI)**	**(95% CI)**	
WBC	51	1.63 to 51.8	0.992	0.95	0.37	− 0.02 (11.1)
× 10^3^/µL				(0.91, 0.99)	(0.06, 1.01)	
RBC	51	2.88 to 6.17	0.939	0.98	0.08	0.01 (2.8)
× 10^6^/µL				(0.91, 1.03)	(− 0.15, 0.41)	
PLT	51	98 to 582	0.911	0.83	25.8	− 0.07 (7.3)
× 10^3^/µL				(0.68, 0.98)	(− 19.3, 63)	
HGB	51	9.7 to 21.1	0.972	0.92	0.89	− 0.03 (2.7)
g/dL				(0.87, 0.99)	(− 0.32, 1.77)	
HCT	51	29 to 64.9	0.925	1.05	− 2.35	0.01 (2.8)
%				(0.96, 1.17)	(− 8.71, 2.94)	
MCV	51	87.7 to 117	0.879	0.81	21.93	0.01 (0.8)
fL				(0.66, 0.97)	(3.99, 36.4)	
RDW	51	14.2 to 21.4	0.875	0.5	7.35	− 0.07 (1.7)
%				(0.43, 0.56)	(6.03, 8.29)	
MCH	51	29.1 to 40.5	0.875	0.86	3.8	− 0.04 (0.7)
pg				(0.78, 0.96)	(0.30, 6.65)	
MCHC	51	30.5 to 36.1	0.122	0.28	22.87	− 0.04 (1)
g/dL				(-0.37, 0.55)	(13.53, 45.38)	
MPV	50	7.1 to 12.1	0.615	0.71	0.89	− 0.22 (2.3)
fL				(0.5, 1)	(− 2, 3.13)	
GRAN#	39	0.4 to 40.8	0.995	0.90	0.19	− 0.08 (NEUT 14.1 EOS 15 BASO 12.4)
× 10^3^/µL				(0.86, 0.93)	(0.02 0.60)	
LYMPH#	36	1.8 to 14.3	0.933	1.14	0.05	0.16 (10.8)
× 10^3^/µL				(0.99, 1.53)	(− 1.18 0.39)	
MONO#	39	0.2 to 5.7	0.908	0.74	0.13	− 0.12 (13.3)
× 10^3^/µL				(0.61, 1.02)	(− 0,12 0.29)	

*BASO*, basophil; *CI*, confidence interval; *EOS*, eosinophil; *HCT*, hematocrit; *HGB*, hemoglobin; *LYMPH*, lymphocyte; *MCHC*, mean corpuscular hemoglobin concentration; *MCH*, mean corpuscular hemoglobin; *MCV*, mean corpuscular volume; *MONO*, monocyte; *NEUT*, neutrophil; *PLT*, platelet; *WBC*, white blood cell; *RBC*, red blood cell; *RDW*, red blood cell distribution width. Desirable limits for inaccuracy (%) have been provided by Aarsand AK, Fernandez-Calle P, Webster C, Coskun A, Gonzales-Lao E, Diaz-Garzon J, Jonker N, Simon M, Braga F, Perich C, Boned B, Marques-Garcia F, Carobene A, Aslan B, Sezer E, Bartlett WA, Sandberg S. The EFLM Biological Variation Database. https://biologicalvariation.eu/ [time of access]

In terms of acceptability, the values relating to slope and intercept sometimes showed slight variability, suggesting possible proportional and systematic errors.

The agreement evaluation, carried out by the Bland–Altman test, showed values of bias < 10% for all parameters, except for MPV, lymphocytes and monocytes (Fig. 1B and Table 3).

In the newborn population, erythroblasts (or NRBC, Nucleated Red Blood Cells), were detected in 23 out of 51 samples analyzed using laboratory instrumentation. Of these 23 samples, only 4 (17.4%) exhibited the L1 flag on the Microsemi CRP LC-767G system, indicating the presence of erythroid precursors, platelet aggregates or abnormal lymphocytes, namely clusters of cells smaller than leukocyte populations. Specifically, in 3 of these 4 samples, leukocytosis was observed with erythroblast percentage in the range of 1–8% while, in the single leukopenic sample with the L1 flag, no appreciable number of erythroblasts was observed on the reference instrumentation. In the remaining 19 cases, in which erythroblasts were detected exclusively during analysis with laboratory instrumentation, 8 had an erythroblast percentage > 10%.

**Fig. 2:**
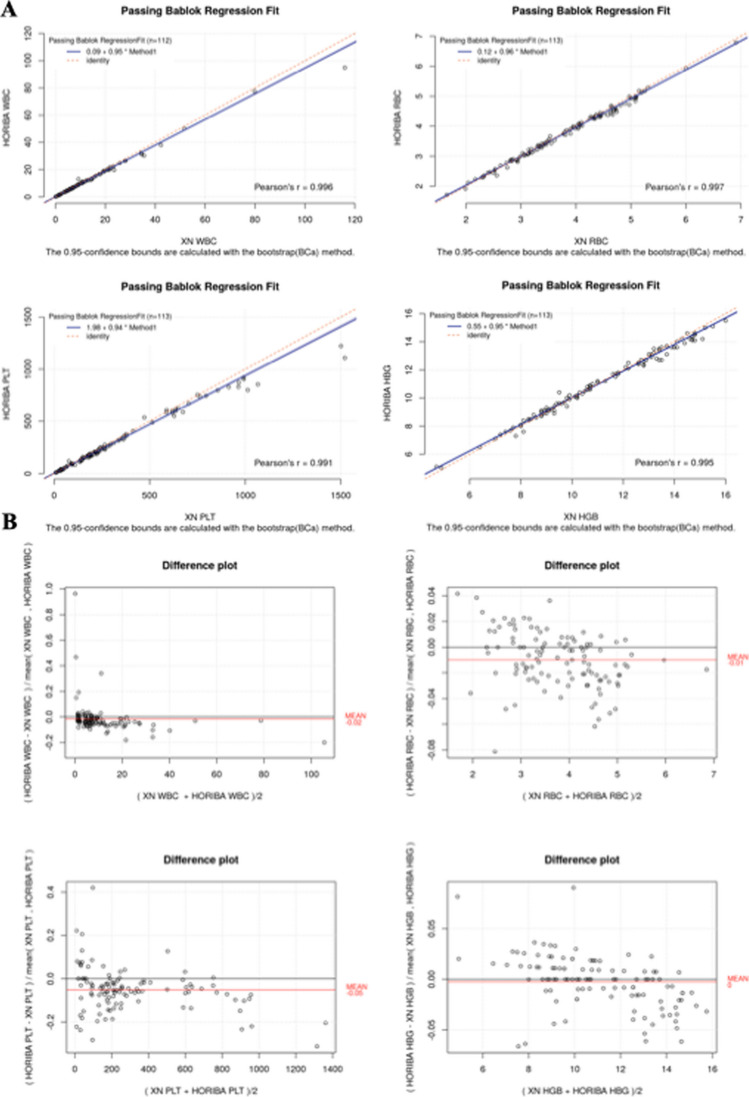
CBC regression analysis and bias plot in adult population. Results of the method comparison study between the Microsemi CRP LC-767G and the Sysmex XN^TM^ hematology analyzers. Graphs indicate Pearson correlation, slope and intercept (**A**), and bias plots (**B**) for the main parameters. WBC, white blood cells; RBC, red blood cells; PLT, platelets; HGB, Hemoglobin

Similar results to those obtained in the newborn population were observed in the adult population, with a better correlation, probably due to a larger sample size (Fig. 2A and Table 4). In particular, it was observed: high correlation (*r* ≥ 0.90) for the parameters WBC, RBC, PLT, HGB, HCT, MCV, RDW, MCH, granulocytes, and lymphocytes; moderate correlation (0.75 < *r* < 0.90) for MPV and low correlation for MCHC (*r* = 0.74) and monocytes (*r* = 0.725).

**Table 4:** Summary of the method comparison study in adult population

Measurand	N	Results range	Correlation coefficient	Slope	Intercept	Mean bias Desirable limits for inaccuracy (%)
			**(*r*)**	**(95% CI)**	**(95% CI)**	
WBC	112	0.07 to 79.8	0.996	0.95	0.08	− 0.02 (11.1)
× 10^3^/µL				(0.94, 0.96)	(0.04, 0.15)	
RBC	113	1.65 to 5.31	0.997	0.96	0.12	− 0.01 (2.8)
× 10^6^/µL				(0.95, 0.98)	(0.06, 0.16)	
PLT	113	8 to 1521	0.991	0.94	1.98	− 0.05 (7.3)
× 10^3^/µL				(0.92, 0.95)	(− 1.12 4.15)	
HGB	113	4.7 to 16	0.995	0.95	0.55	0 (2.7)
g/dL				(0.93, 0.96)	(0.37, 0.72)	
HCT	113	15.6 to 47.1	0.992	1	0	0 (2.8)
%				(0.97, 1.02)	(− 1.33, 0.43)	
MCV	113	62.3 to 111	0.967	0.94	6.49	0.01 (0.8)
fL				(0.88, 1)	(1.51, 11.89)	
RDW	112	12 to 25.7	0.932	0.58	6.31	0 (1.7)
%				(0.53, 0.62)	(5.65, 7)	
MCH	113	17.1 to 37.7	0.986	0.97	1.05	0.01 (0.7)
pg				(0.94, 1)	(0.2, 1.9)	
MCHC	113	27.9 to 36.3	0.74	0.6	13.02	0 (1)
g/dL				(0.48, 0.73)	(8.68, 16.99)	
MPV	105	6.9 to 13.6	0.882	0.7	1.05	− 0.23 (2.3)
fL				(0.64, 0.75)	(0.46 1.59)	
GRAN#	110	0.3 to 40.9	0.986	0.95	0.07	0 (NEUT 14.1 EOS 15 BASO 12.4)
× 10^3^/µL				(0.93, 0.96)	(− 0.008 0.12)	
LYMPH#	110	0.1 to 73.4	0.998	0.94	0.18	0.17 (10.8)
× 10^3^/µL				(0.91, 0.99)	(0.13 0.23)	
MONO#	108	0.1 to 9.5	0.725	0.67	0.003	− 0.33 (13.3)
× 10^3^/µL				(0.58, 0.78)	(− 0,05 0.05)	

*BASO*, basophil; *CI*, confidence interval; *EOS*, eosinophil; *HCT*, hematocrit; *HGB*, hemoglobin; *LYMPH*, lymphocyte; *MCHC*, mean corpuscular hemoglobin concentration; *MCH*, mean corpuscular hemoglobin; *MCV*, mean corpuscular volume; *MONO*, monocyte; *NEUT*, neutrophil; *PLT*, platelet; *WBC*, white blood cell; *RBC*, red blood cell; *RDW*, red blood cell distribution width. Desirable limits for inaccuracy (%) have been provided by Aarsand AK, Fernandez-Calle P, Webster C, Coskun A, Gonzales-Lao E, Diaz-Garzon J, Jonker N, Simon M, Braga F, Perich C, Boned B, Marques-Garcia F, Carobene A, Aslan B, Sezer E, Bartlett WA, Sandberg S. The EFLM Biological Variation Database. https://biologicalvariation.eu/ [time of access]

In terms of acceptability, as for the newborn population, the values relating to slope and intercept sometimes showed slight variability, suggesting possible proportional and systematic errors.

The agreement evaluation, carried out by the Bland–Altman test, showed values of bias < 10% for all parameters, except for MPV, lymphocytes, and monocytes (Fig. 2B and Table 4), as for the newborn population.

In the adult population, the L1 Flag occurred in 10 out of 113 cases, but only one was confirmed by the Sysmex XN-9100^TM^ laboratory instrumentation.

### Method comparison: CRP results

**Fig. 3:**
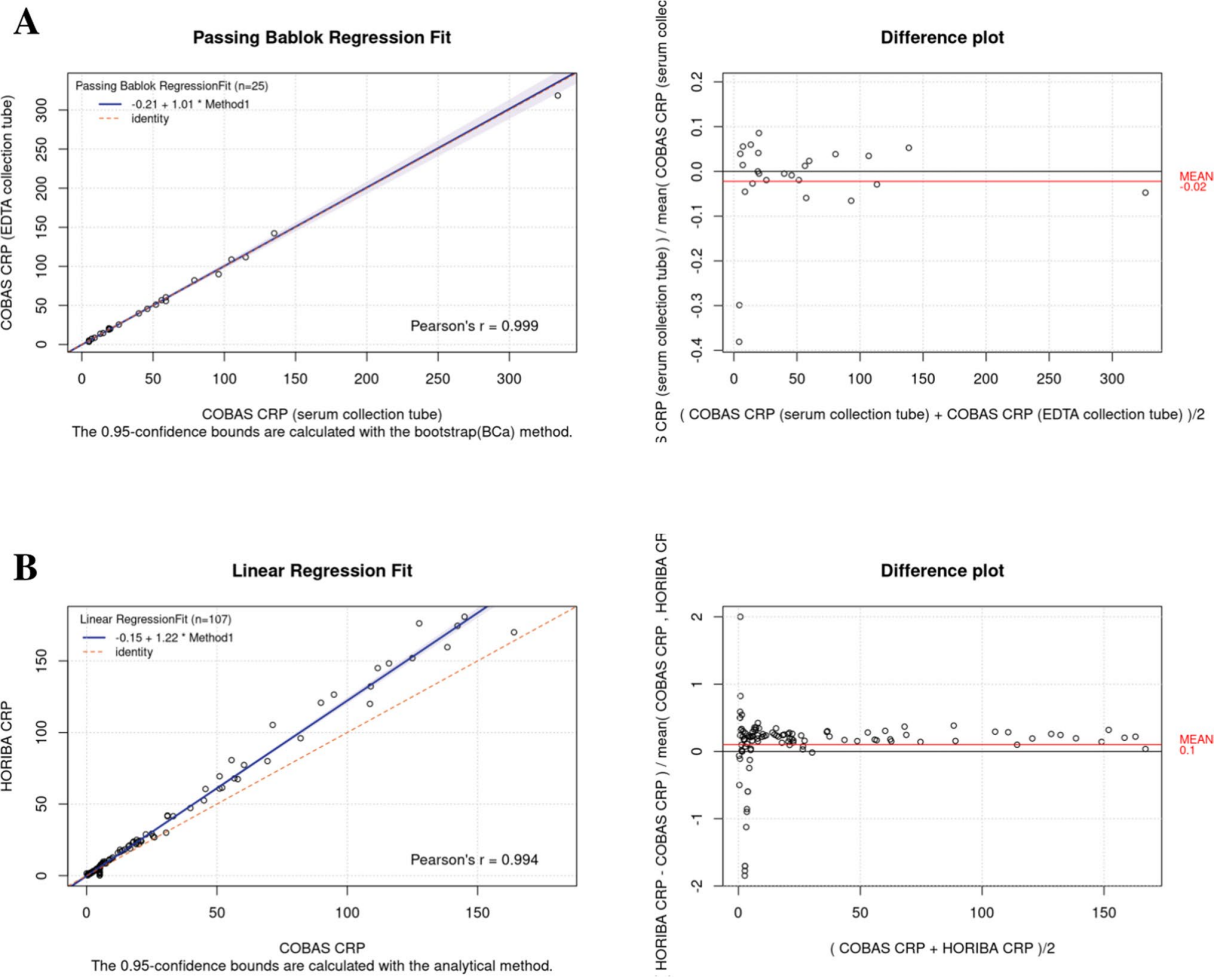
CRP regression analysis and bias plot in adult population. Results of the different matrices effect (**A**) and results of the method comparison study (**B**) between the Microsemi CRP LC-767G and the Roche Cobas® c702. Graphs indicate Pearson correlation, slope and intercept (left panel), and bias plot (right panel)

The comparison between the different matrices (whole blood vs serum samples) showed an excellent agreement (*r* = 0.999), with intercept and slope values of − 0.21 (95% CI, − 1.50, 0.63) and 1.005 (95% CI, 0.96, 1.04), respectively, and a mean bias of 2% (Fig. 3A).

The instrumental comparison results, performed on the adult population, showed an optimal agreement, (*r* = 0.994), with intercept and slope values of − 0.152 (95% CI, − 1.41, 1.10) and 1.22 (95% CI, 1.2, 1.25), respectively, and the mean bias is 10% (Fig. 3B).

## Discussion

New technologies are increasingly being developed in healthcare to simplify decision making, enable patient-centered care, improve overall community resilience, and reduce costs and resource use, by providing more efficient services and more effective care. POCT systems could satisfy all these needs. The benefits of reducing TAT and sampling volumes are clear, maintaining accuracy and sensitivity comparable to laboratory methods and even cost savings in some cases [10, 11].

The SARS-CoV-2 pandemic has given a strong effort towards research on microdevices by authorizing and implementing the spread of POCT not only in local settlements, but also in hospital departments with specific requirements such as emergency rooms and critical care settings.

The implementation of a new POCT instrument in the clinical diagnostic pathway, managed by a multidisciplinary group according to the ISO 15189:2022 accreditation standards, involves a series of key steps, some of which precede the introduction into clinical practice, such as performance validation of the instrument and staff training, while others (quality controls, continuous staff training, and constant improvement) come into play once the new test has been integrated into routine practice [12, 13].

This paper evaluated the performance of the Microsemi CRP LC-767G POCT system, verifying the interchangeability of its results with those of reference systems.

The method comparison shows that the Microsemi CRP LC-767G system provides hematology results comparable to those obtained with the Sysmex XN-9100™ system, over a wide range of measurements, including highly pathological samples.

Considering the newborn population (*N* = 51), the results show a good correlation between the Microsemi CRP LC-767G analyzer and the Sysmex XN-9100™. A high correlation (*r* ≥ 0.90) was observed for the key hematological parameters of the basic blood count (WBC, RBC, PLT, HGB, and HCT) and also for the three leukocyte populations (granulocytes, lymphocytes, and monocytes). The low correlation for the MCHC parameter is due to the fact that, in both instruments, the value is calculated from a ratio of two highly correlated erythrocyte parameters (MCH and MCV or HGB and HCT), leading to a small range of MCHC values; these ratios mathematically amplify any small system-specific differences in each of the two parameters, thus impairing the correlation in the calculated parameter.

In terms of acceptability, the slope and intercept values sometimes exhibit variability, suggesting potential slight systematic errors. No proportional or constant differences were observed for the measurement of RBC, HGB, HCT, PLT, MPV, lymphocytes, and monocytes that would impede interchangeability between the two measurement systems.

Considering the adult population (*N* = 113), the results of regression and concordance analysis are in line with those observed in newborn cases, however showing a higher correlation (*r* > 0.98) for hematological parameters such as WBC, RBC, PLT, HGB, HCT, MCH, granulocytes and lymphocytes, probably due to the larger sample size.

The C-reactive protein results obtained on adult samples and analyzed on Microsemi CRP LC-767G and Roche Cobas® c702 showed a high correlation (*r* = 0.994). The role of different matrices (whole blood vs serum) was studied on 25 samples and the results showed excellent correlation (*r* = 0.999). A constant and proportional systematic error was observed between the two instrumentations, with a generally acceptable bias (10%).

Regarding the instrument management, the Microsemi CRP LC-767G system requires testing of at least two internal quality control levels every 24 h, before measuring a sample, to confirm that the instrument accuracy is maintained. This practice is in line with existing protocols for other decentralized laboratory equipment and is already familiar to care unit staff. If even one level of control produces unacceptable results, the instrument prevents analysis of the sample, requiring further attempts to process the control or the intervention of laboratory personnel dedicated to POCT instrumentation.

The Microsemi CRP LC-767G system can therefore represent an alternative but effective testing approach outside the laboratory, particularly in NICU, to reduce the impact of pre-analytical errors on newborn samples.

The advantages could rely in terms of practical purposes and analytical solutions.

Time is a critical factor and delays in the transport of samples significantly impact diagnosis and patient treatment. These difficulties emerge especially when samples are not appropriate and therefore acceptable for laboratory testing, rising the reluctance of healthcare personnel to expose fragile patients to repeated blood sampling.

The blood volume coming from venipuncture is critical in premature newborns (gestational age < 28 weeks at birth): 1 ml of blood of a 0.5 kg premature newborn represents approximately 2.5% of the total blood volume. Therefore, the use of small volume in POCT and microsystem devices is ideal for these patients.

In hematological analysis, another critical issue concerns the frequent activation of the coagulation cascade, even in presence of an anticoagulant, often associated with the time between sample collection and analysis. This critical issue is more frequent in samples with difficult collection and very low volume, such as those from the NICU. In our study, 22.8% of the analyzed samples were coagulated. The identification of clotted samples can only occur through visual inspection by laboratory personnel, often using a micropipette to assess the blood fluidity and viscosity. This technical step is crucial and cannot be replaced, not even with decentralized instrumentation.

About the analytical performances, the most critical issue that may require careful review of internal procedures related to the NICU is the potential overestimation of total white blood cells due to the possible presence of erythroblasts in the peripheral blood of newborns. Sysmex XN-9100™ has a specific channel for the discrimination and quantification of erythroblasts from total white blood cells, whereas, in the Microsemi LC-767G system, nucleated red cell precursors are included in the leukocyte count and samples that report this interference are labeled with flag L1. However, as observed in our newborn population, L1 flag appears unreliable, with only 17% of NRBC samples confirmed via POCT instrumentation.

In Italy, perinatal care centers are organized on three levels considering the severity of pregnancy risk. Even the first level centers (physiological pregnancy, low and intermediate obstetric risk, and healthy newborn) should be equipped with all the services necessary for adequate assistance, including the laboratory, with the possibility of having rapid responses (30') for emergency tests. Moreover, among the structural standards of NICUs, the presence of a suitable space for small laboratory activities is highly desirable. Since inadequate sample collection and transport can invalidate analytes quantification (coagulated or hemolyzed sample), the availability of reliable and accurate Point-of-care testing has an important impact to assure the reduction of turnaround times and rapid patient management in clinical settings where it is important to make quick decisions.

Since POCT analyses are performed especially by non-laboratory healthcare personnel, the challenge with POCT is the compliance with quality assurance programs. Proficiency testings should be approached in order to implement a safe and proficient use of CBC analysis by POCT devices in the pediatric hospital.

As addressed in this work, the advances in the technology of POCT systems have achieved performances comparable to laboratory instrumentation and therefore the next implementation will have to include a network between decentralized testing and territorial/hospital reference laboratories. In order to improve patient safety, the appropriate tools and parameters of risk management for each phase of the entire analytical process should be defined according to international guidelines such as EP 23A (Laboratory Quality Control Based on Risk Management) by the Institute of Clinical and Laboratory Standards (CLSI).

## Conclusions

The use of the Microsemi CRP LC-767G system is aimed at all healthcare personnel, for an easy-to-use and rapid testing of hematological parameters and CRP. It is suitable for emergency and critical units such as NICU, reducing the risk of samples noncompliant with analyses due to the time between collection and analysis.

As demonstrated by the results of our study, the performance of the Microsemi CRP LC-767G analyzer is comparable to that of the reference instrumentation at the analytical level. However, it remains crucial to train healthcare personnel to assess sample analysis compliance and identify any pre-analytical issues.

Nowadays, POCT devices have improvements in regulatory compliance and quality assurance standards, that must be under the laboratory staff management and control.

## Supplementary Information

Below is the link to the electronic supplementary material.

ESM 1 (PNG 472 KB)

Figure 1S-2S. CBC bias plot in newborn population. Results of the method comparison study between the Microsemi CRP LC-767G and the Sysmex XN^TM^ hematology analyzers. Graphs indicate bias plots for all parameters. The overall bias was calculated as the values on the axis [Reference method vs. the difference between two measurements]. BASO, basophil; EOS, eosinophil; HCT, hematocrit; HGB, hemoglobin; LYMPH, lymphocyte; MCHC, mean corpuscular hemoglobin concentration; MCH, mean corpuscular hemoglobin; MCV, mean corpuscular volume; MONO, monocyte; NEUT, neutrophil; PLT, platelet; WBC, white blood cell; RBC, red blood cell; RDW, red blood cell distribution width (TIF 971 KB)

ESM 2 (PNG 277 KB)

High Resolution Image (TIF 578 KB)

ESM 3 (PNG 454 KB)

Figure 3S-4S. CBC bias plot in adult population. Results of the method comparison study between the Microsemi CRP LC-767G and the Sysmex XN^TM^ hematology analyzers. Graphs indicate bias plots for all parameters. The overall bias was calculated as the values on the axis [Reference method vs. the difference between two measurements]. BASO, basophil; EOS, eosinophil; HCT, hematocrit; HGB, hemoglobin; LYMPH, lymphocyte; MCHC, mean corpuscular hemoglobin concentration; MCH, mean corpuscular hemoglobin; MCV, mean corpuscular volume; MONO, monocyte; NEUT, neutrophil; PLT, platelet; WBC, white blood cell; RBC, red blood cell; RDW, red blood cell distribution width (TIF 940 KB)

ESM 4 (PNG 294 KB)

High Resolution Image (TIF 618 KB)

ESM 5 (PNG 277 KB)

Figure 5S. CRP bias plot in adult population. Results of the different matrices effect (left panel) and results of the method comparison study (B) between the Microsemi CRP LC-767G and the Roche Cobas® c702. Graphs indicate bias plots. The overall bias was calculated as the values on the axis [Reference method vs. the difference between two measurements] (TIF 303 KB)

## Data Availability

No datasets were generated or analysed during the current study.

## Abbreviations

CBC: Complete blood count
CI: Confidence interval
CLSI: Clinical and Laboratory Standards Institute
CRP: C-reactive protein
EDTA-2K: Ethylenediamine tetraacetic acid dipotassium
GRA: Granulocytes
HCT: Hematocrit
HGB: Hemoglobin
ISO: International Organization for Standardization
LYM: Lymphocytes
MCH: Mean corpuscolar hemoglobin
MCHC: Mean corpuscolar hemoglobin concentration
MCV: Mean corpuscolar volume
MON: Monocytes
MPV: Mean platelet volume
NICU: Neonatal intensive care unit
NRBC: Nucleated red blood cells
PLT: Platelets
POCT: Point-of-care testing
RBC: Red blood cells
RDW: Red blood cell distribution width
SARS-CoV-2: Severe acute respiratory syndrome coronavirus 2
TAT: Turnaround time
WBC: White blood cells
