# Supplementary figures and images for: Rapid complete blood count and C-reactive protein determination with the Horiba Microsemi analyzer: the experience in neonatal intensive care unit of Careggi University Hospital
Source: Eur J Pediatr. 2024 Aug 15;183(10):4477–90. doi: 10.1007/s00431-024-05695-0 (PMC11413159; doi:10.1007/s00431-024-05695-0)

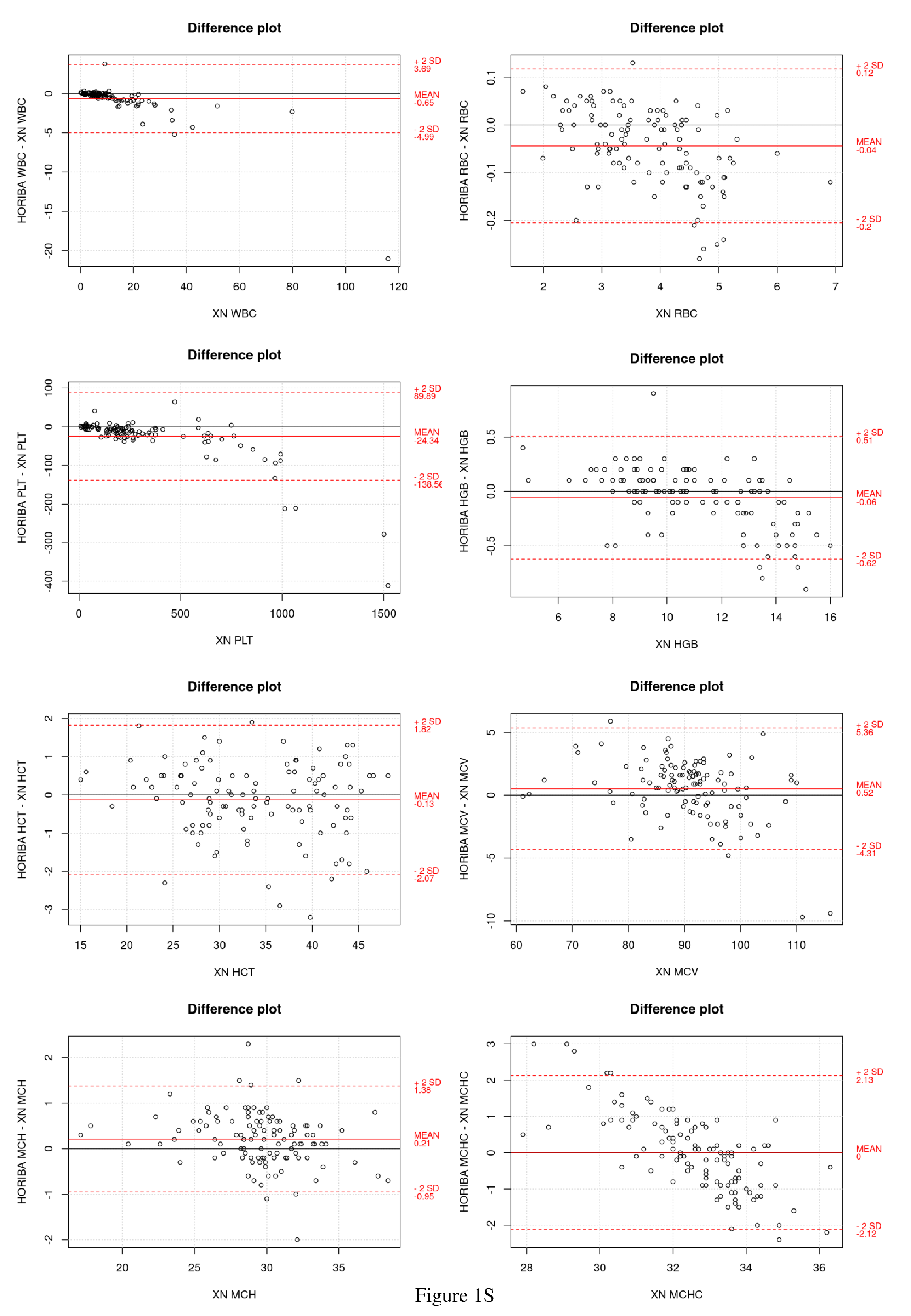

Supplement: Supplementary file 1 — (PNG 472 KB) [file 431_2024_5695_Fig4_ESM.png]

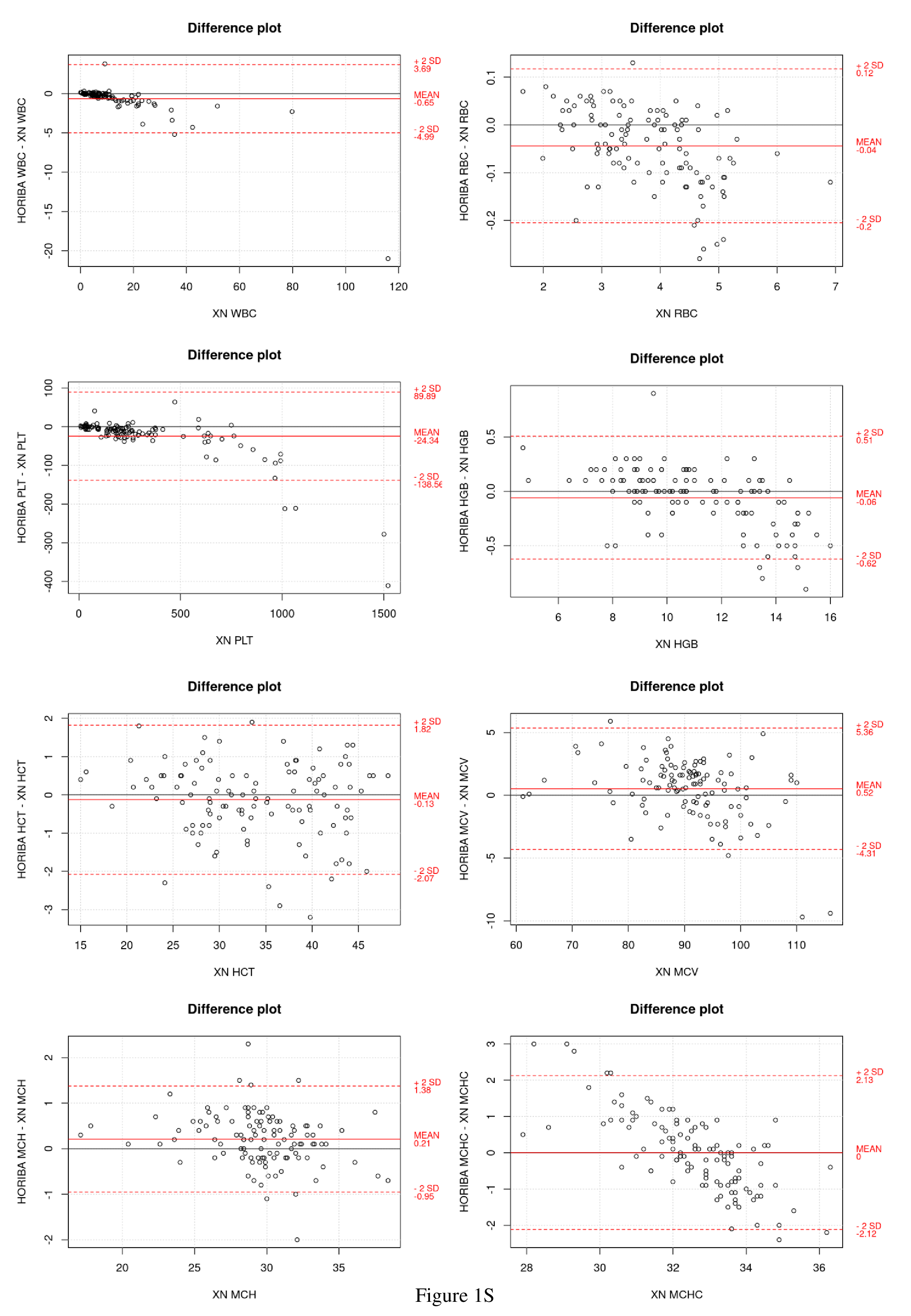

Supplement: Supplementary file 2 — Figure 1S-2S. CBC bias plot in newborn population. Results of the method comparison study between the Microsemi CRP LC-767G and the Sysmex XNTM hematology analyzers. Graphs indicate bias plots for all parameters. The overall bias was calculated as the values on the axis [Reference method vs. the difference between two measurements]. BASO, basophil; EOS, eosinophil; HCT, hematocrit; HGB, hemoglobin; LYMPH, lymphocyte; MCHC, mean corpuscular hemoglobin concentration; MCH, mean corpuscular hemoglobin; MCV, mean corpuscular volume; MONO, monocyte; NEUT, neutrophil; PLT, platelet; WBC, white blood cell; RBC, red blood cell; RDW, red blood cell distribution width (TIF 971 KB) [file 431_2024_5695_MOESM1_ESM.tiff]

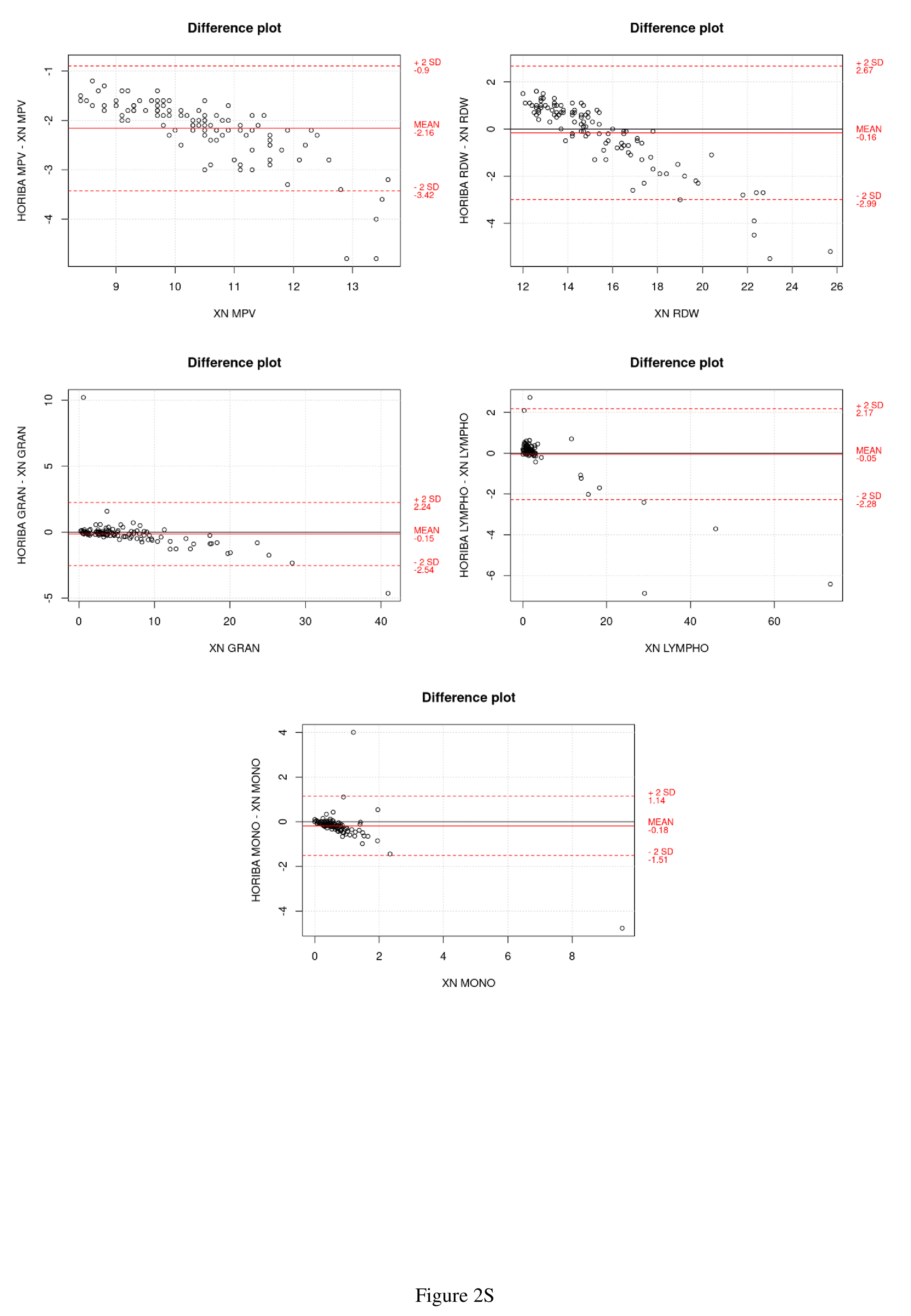

Supplement: Supplementary file 3 — (PNG 277 KB) [file 431_2024_5695_Fig5_ESM.png]

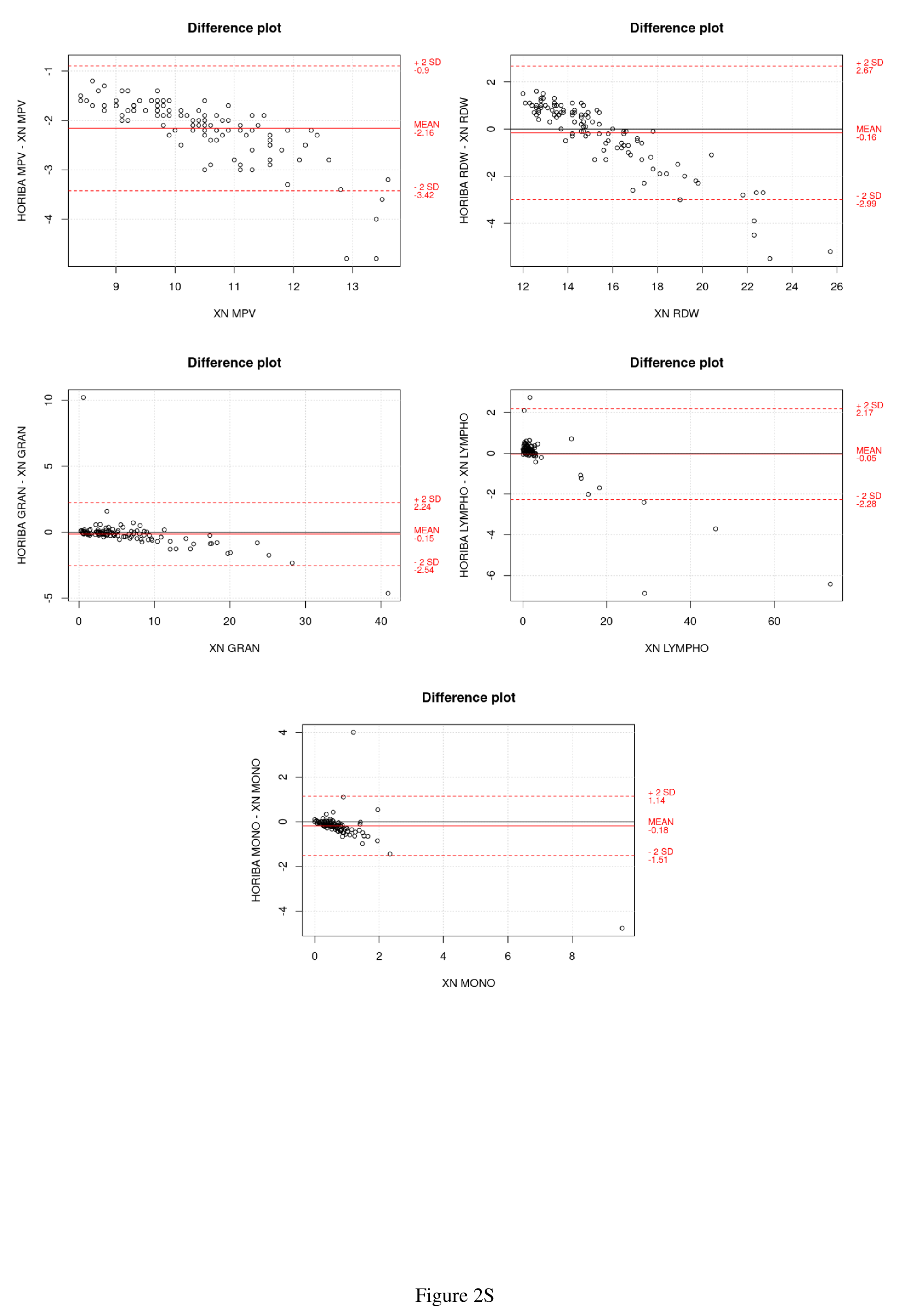

Supplement: Supplementary file 4 — High Resolution Image (TIF 578 KB) [file 431_2024_5695_MOESM2_ESM.tiff]

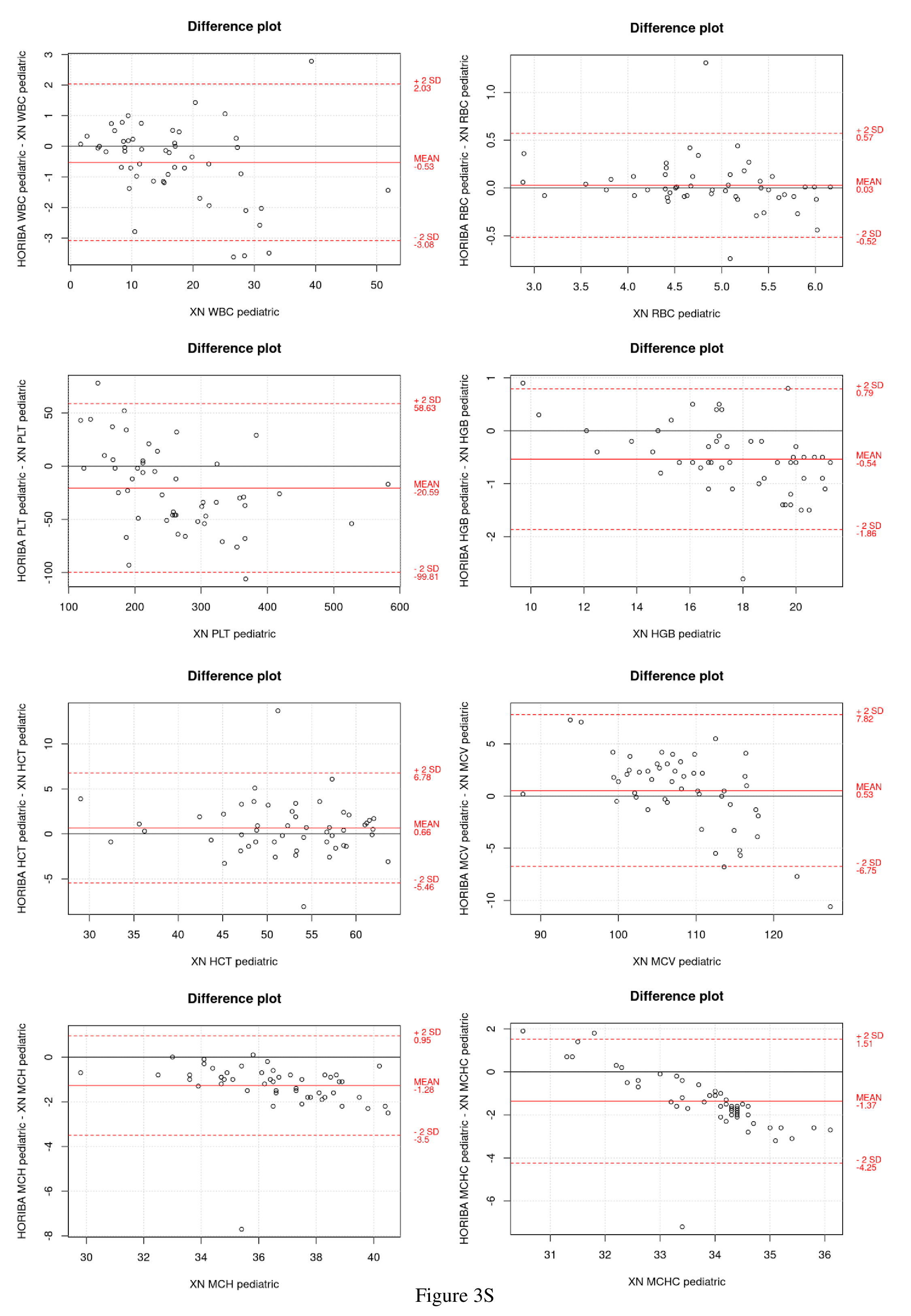

Supplement: Supplementary file 5 — (PNG 454 KB) [file 431_2024_5695_Fig6_ESM.png]

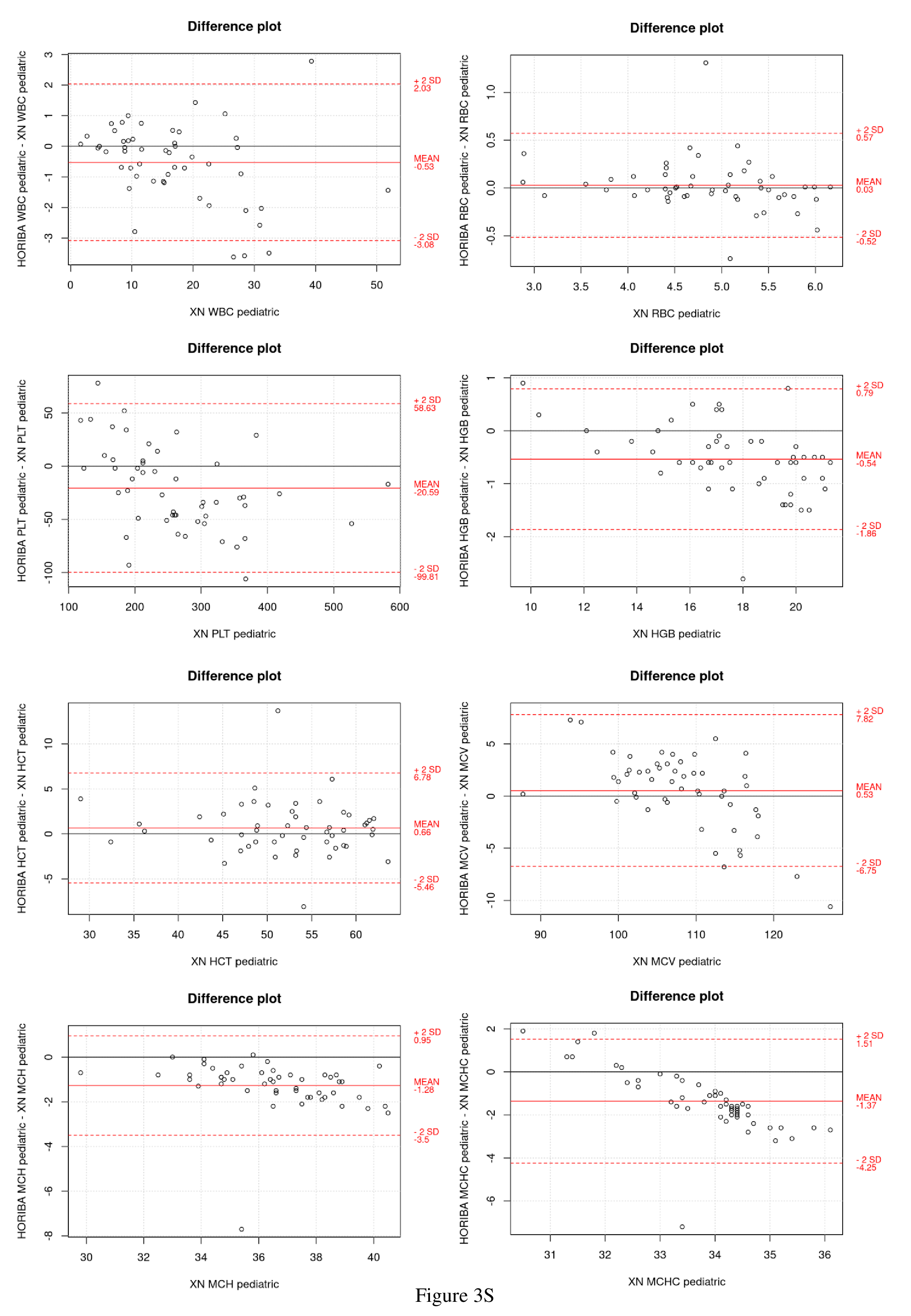

Supplement: Supplementary file 6 — Figure 3S-4S. CBC bias plot in adult population. Results of the method comparison study between the Microsemi CRP LC-767G and the Sysmex XNTM hematology analyzers. Graphs indicate bias plots for all parameters. The overall bias was calculated as the values on the axis [Reference method vs. the difference between two measurements]. BASO, basophil; EOS, eosinophil; HCT, hematocrit; HGB, hemoglobin; LYMPH, lymphocyte; MCHC, mean corpuscular hemoglobin concentration; MCH, mean corpuscular hemoglobin; MCV, mean corpuscular volume; MONO, monocyte; NEUT, neutrophil; PLT, platelet; WBC, white blood cell; RBC, red blood cell; RDW, red blood cell distribution width (TIF 940 KB) [file 431_2024_5695_MOESM3_ESM.tiff]

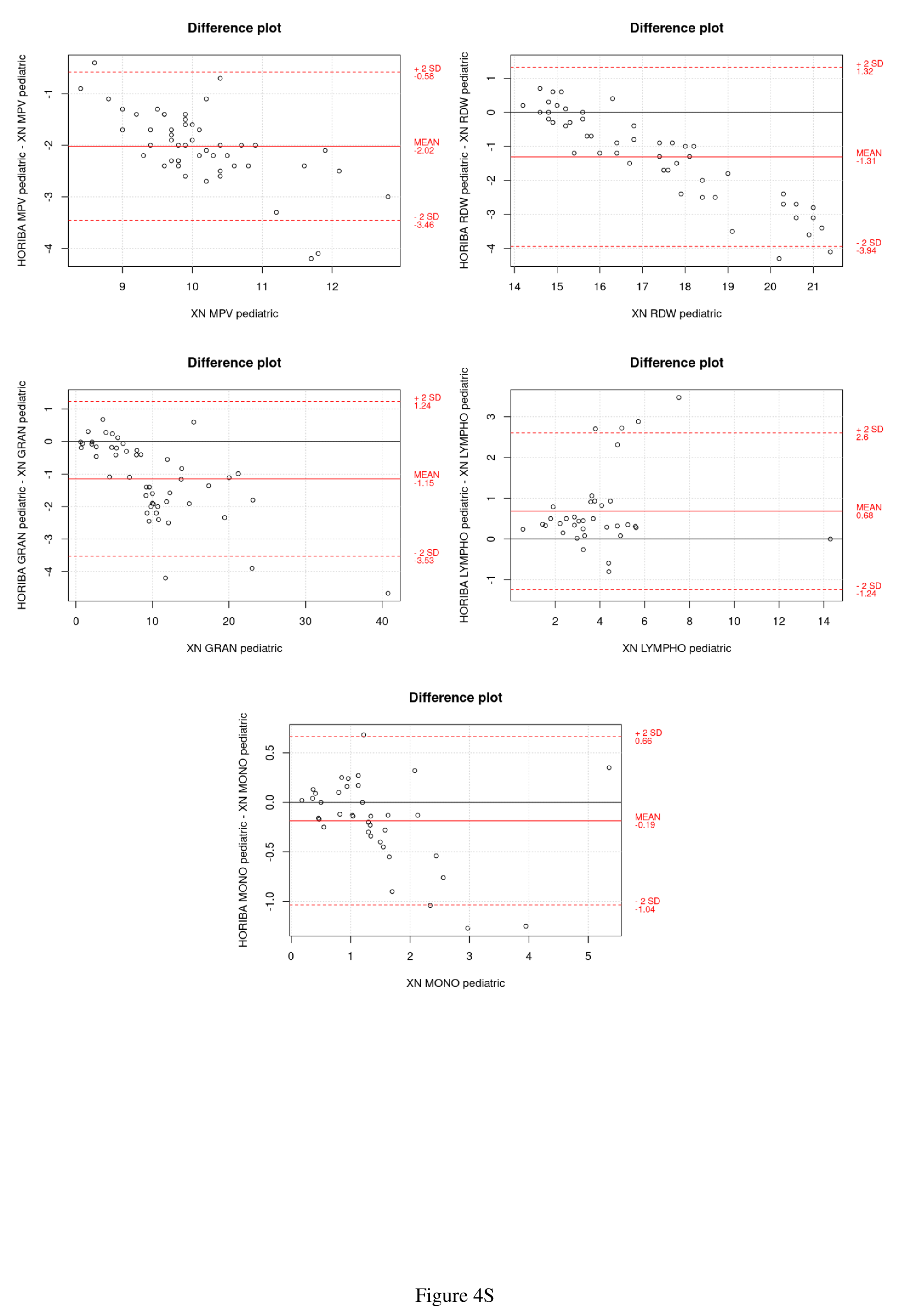

Supplement: Supplementary file 7 — (PNG 294 KB) [file 431_2024_5695_Fig7_ESM.png]

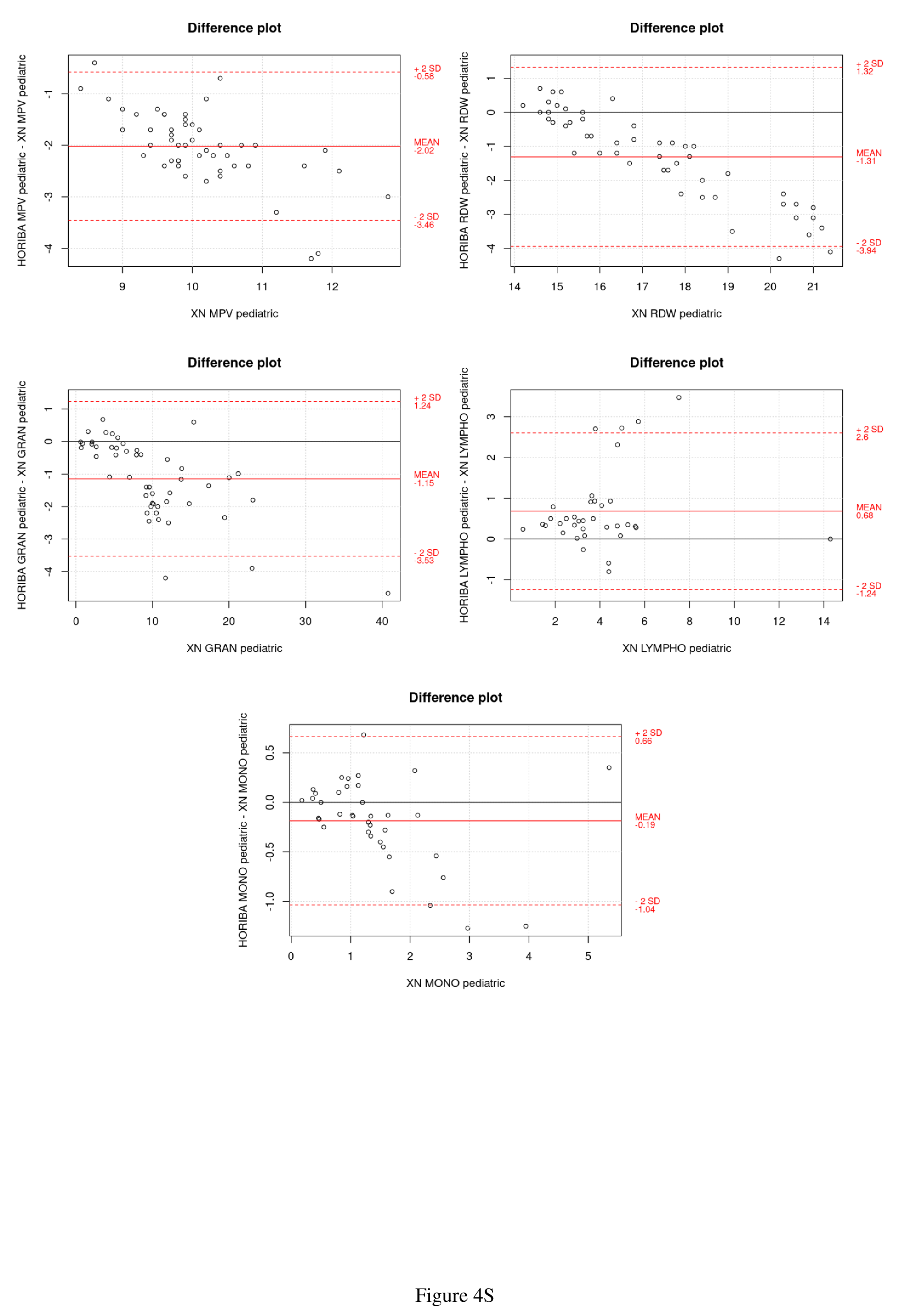

Supplement: Supplementary file 8 — High Resolution Image (TIF 618 KB) [file 431_2024_5695_MOESM4_ESM.tiff]

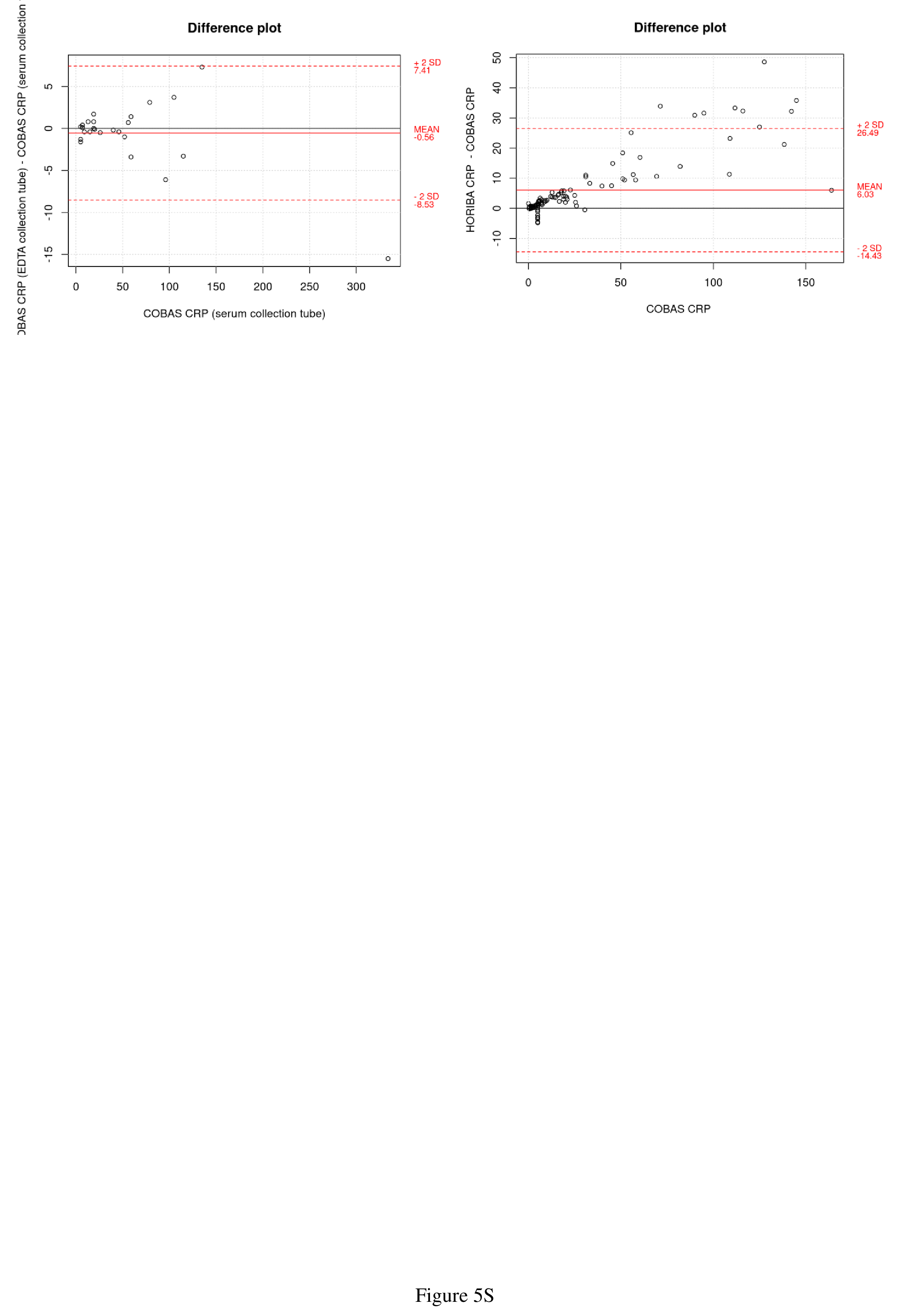

Supplement: Supplementary file 9 — (PNG 277 KB) [file 431_2024_5695_Fig8_ESM.png]

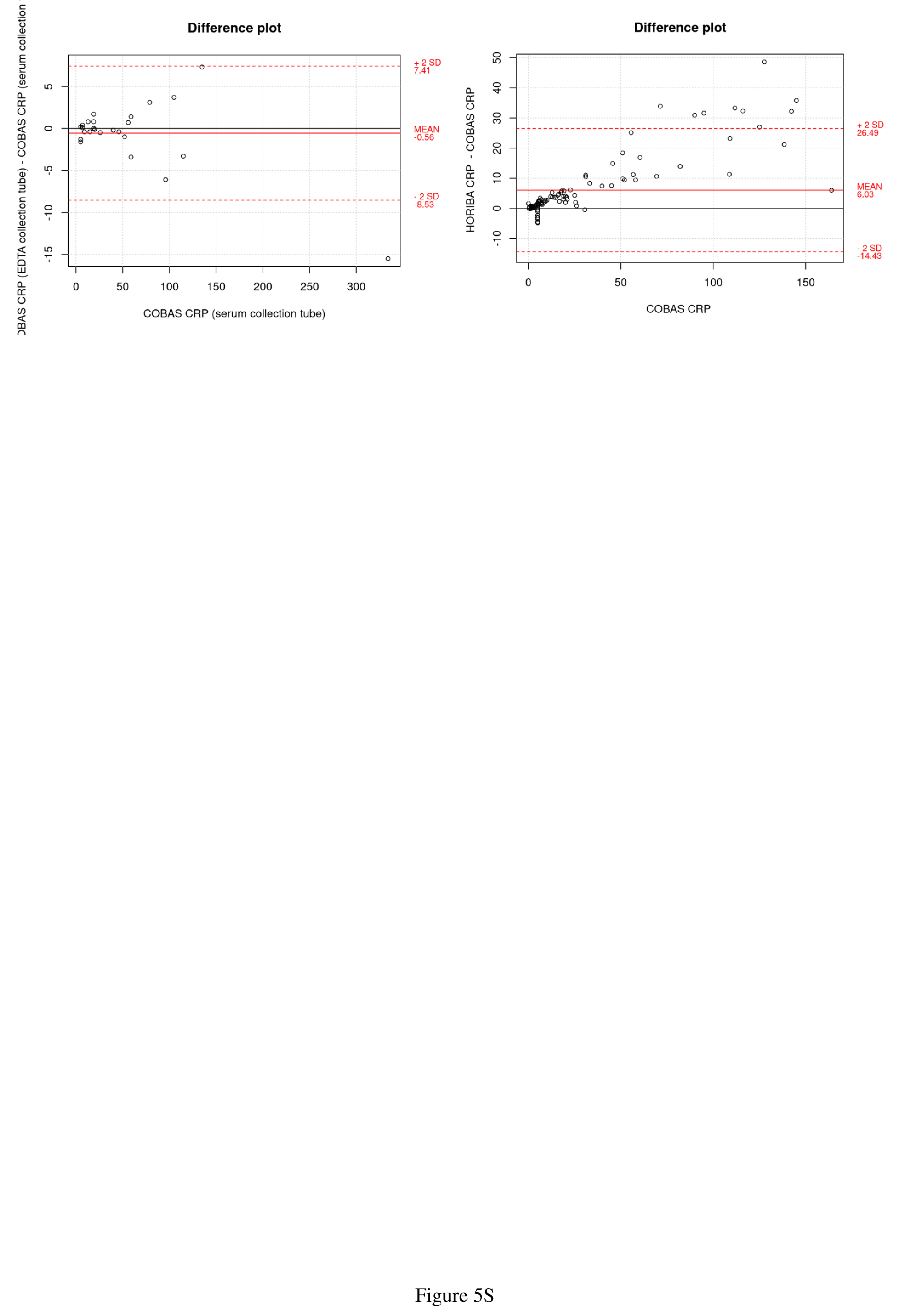

Supplement: Supplementary file 10 — Figure 5S. CRP bias plot in adult population. Results of the different matrices effect (left panel) and results of the method comparison study (B) between the Microsemi CRP LC-767G and the Roche Cobas® c702. Graphs indicate bias plots. The overall bias was calculated as the values on the axis [Reference method vs. the difference between two measurements] (TIF 303 KB) [file 431_2024_5695_MOESM5_ESM.tiff]
